# Stomach Carcinoma Presenting with a Synchronous Liver Cancer: A Case Report and Literature Review

**DOI:** 10.1155/2014/970293

**Published:** 2014-09-22

**Authors:** Yong Wang, Xiao-ting Wu

**Affiliations:** Department of Gastrointestinal Surgery, West China Hospital, Sichuan University, 37 Guo Xue Road, Chengdu, Sichuan 610041, China

## Abstract

Multiple primary malignant neoplasms are two or more malignancies in an individual without any relationship between the tumors. Multiple primary malignancies are relatively rare but have increased in recent decades. Two cancers are commonly observed among those with multiple primary malignancies, but two malignancies of stomach and liver are relatively rare to be reported. Mechanisms of the tumors were unclear; we described a patient who had stomach carcinoma presenting with a synchronous liver cancer and investigated his family history; we suggest that family history may be a key risk factor and early detection for additional primary malignancies should be needed for patients who had specific cancer history in their pedigree. Early diagnosis may be the key risk factor affecting prognosis.

## 1. Introduction

Warren and Gates first described the phenomenon of multiple primary malignant tumors in a single patient [1]. Multiple primary malignant neoplasms, without any relationship between the tumors, are two or more malignancies in an individual [2]. Multiple primary malignancies, being the result of the development of medical skills in diagnostic and therapeutic strategies, new carcinogens' possible effect from the industrial environment and longer life span making primary tumor more likely to develop, are relatively rare but have increased in recent decades [3–5]. Mechanisms of multiple primary malignancies were unclear, which could be attributable to radiotherapy [6]. Two cancers are commonly observed among those with multiple primary malignancies, but two malignancies of stomach and liver are relatively rare to be reported. There is few similar case reports in the literature describing the phenomenon. We present a case of a patient with two cancers of stomach and liver, who had a family history of cancer as well as a history of hepatitis B.

## 2. Case report

### 2.1. Patient

**Figure 1:**
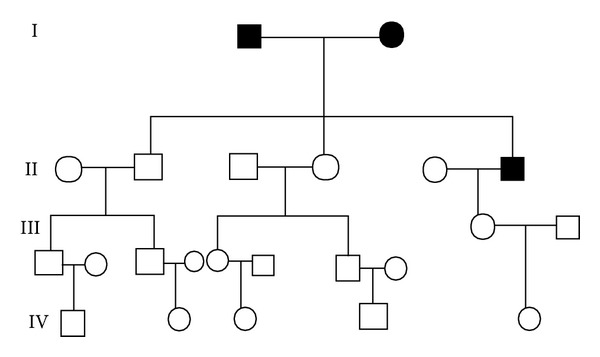
Specific cancer history was recorded on his pedigree; his father died of hepatic cancer and his mother died from lung carcinoma (●  ■ represents one who had cancer).

**Figure 2:**
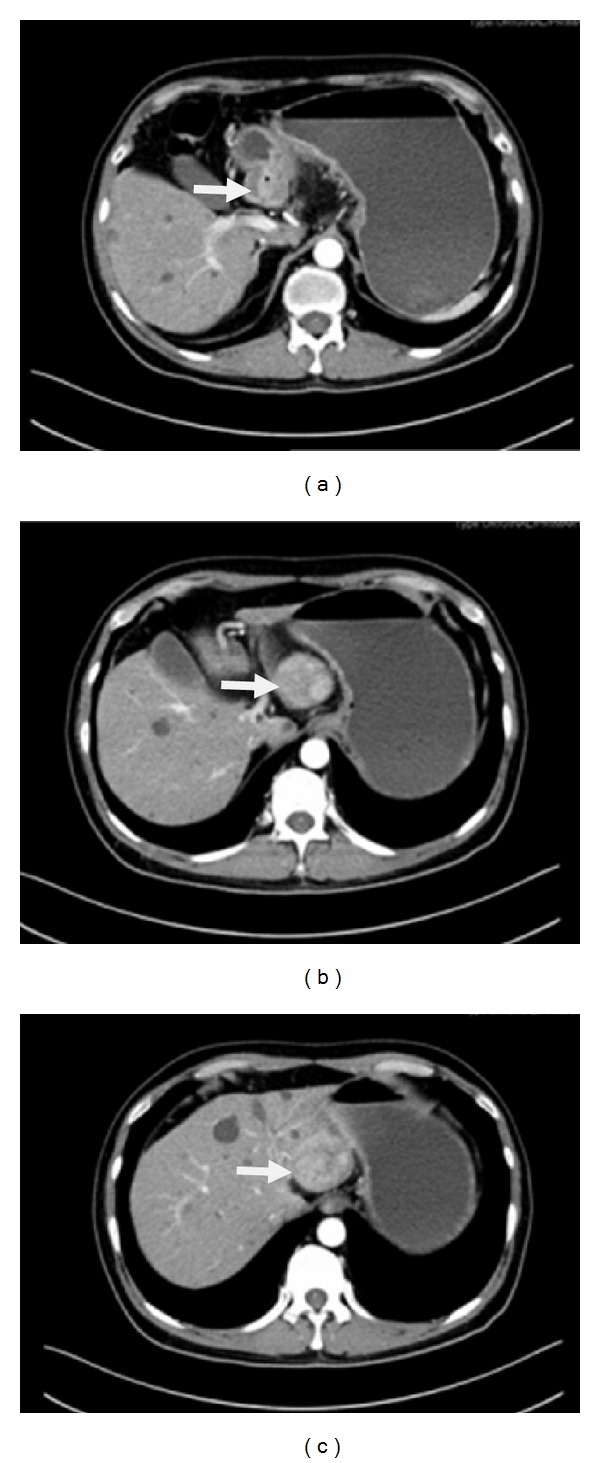
An irregularly thickened and rigid wall of gastric antrum which was able to be enhanced; a mass in the left hepatic lobe, which was measured 5.0 × 4.4 cm and could be partially enhanced.

**Figure 3:**
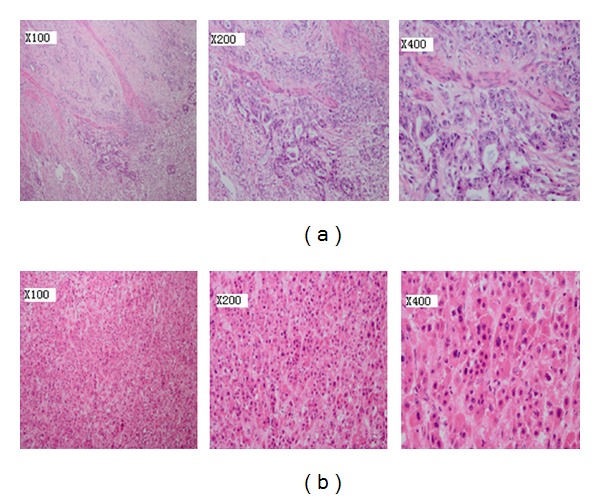
(a) A microscopic examination reveals stomach poorly differentiated adenocarcinoma (hematoxylin and eosin, ×100, ×200, ×400). (b) Primary hepatocellular carcinoma is described by a microscopic examination (hematoxylin and eosin, ×100, ×200, and ×400).

A 58-year-old chronic smoker, a nonalcoholic male was admitted to our hospital due to one-month history of progressive abdominal discomfort in the left upper quadrant associated with nausea, loss of weight, and loss of appetite especially after eating something. Specific cancer history was recorded in his pedigree (Figure 1); his father died of hepatic cancer and his mother died from lung carcinoma as well as his history of hepatitis B. His grandfather and grandmother had no specific cancer history. A computed tomography scan of the abdomen revealed an irregularly thickened and rigid wall of gastric antrum which was able to be enhanced by enhanced computed tomography, and there was a mass in the left hepatic lobe, which was measured 5.0 × 4.4 cm and could be partially enhanced (Figure 2). There was a deep concave ulcer (measured 2 × 2 cm) in gastric antrum near the pylorus and gastric antrum mucosa was thickening and rigid through gastroscope. Endoscopic biopsy from the stomach revealed differentiated tubular adenocarcinoma. Alpha-fetoprotein (AFP) level was 1.63 ng/mL, and carcinoembryonic antigen was 1.22 ng/mL. Radical gastrectomy for cancer as well as liver tumor resection was performed for the patient; the operation was very successful. During the operation, we can see that the tumor was in the anterior wall of the gastric antrum and lesser curvature, measured 5 × 4 × 3 cm in diameter, and visually had invasion into the liver, the stomach ligament, and the pancreatic capsule. There were multiple gastric lymph nodes grown surrounding the stomach, and there was a tumor in hepatic left lateral lobe. The mass was approximately 5 × 4 cm in diameter and it was tough, clear with the surrounding. The pathology demonstrates hepatocellular carcinoma; stomach poorly differentiated adenocarcinoma (signet ring cell carcinoma) as well as metastatic lymphadenopathy (Figures 3(a) and 3(b)). The patient received chemotherapy a month after surgery, and Alpha-fetoprotein (AFP) level, carcinoembryonic antigen, and liver function are normal in the follow-up after one year.

## 3. Discussion

Multiple primary malignant neoplasms are two or more malignancies in a single patient without any relationship between the tumors. Multiple primary malignant tumors can become synchronous or metachronous depending on the interval between their diagnosis. Synchronous cancers are diagnosed simultaneously or within an interval of about 6 month, and metachronous cancers are secondary cancers that developed more than 6 month after the diagnosis and treatment of primary cancers [7]. The criteria of multiple primary malignant tumors included the following: (1) each tumor must present a definite picture of malignancy; (2) each tumor must be histologically distinct; (3) the possibility that one is a metastasis of another must be excluded [1, 8]. The majority of multiple primary cancers may occur as a result of random chance [9]. The mechanisms and risk of multiple primary cancers are unknown, which could be attributable to intense exposure to carcinogens, the effects of chemotherapy and/or radiotherapy [10, 11], and the influence of genetic susceptibility, genetic instability, and longer average life span [12, 13]. Carcinogenic insults, such as tobacco and alcohol, may increase the likelihood of multiple independent malignant foci developing in the mucosa epithelium. In addition, smoking and family history may be the key risk factors of multiple primary malignant neoplasms [14, 15]. Some common points can be described in reviews of the literature about multiple primary malignancies. First, the Japanese population appears to have a higher likelihood of developing multiple primary cancers. Yamamoto et al. reported that 15 to 20% of Japanese patients with colorectal carcinoma developed multiple primary cancers [13]. This may be caused by genetic susceptibility, longer average life span, or medical advances in chemotherapy and radiotherapy. Second, most patients with multiple primary cancers are geriatric. Third, smoking-related cancers, prostate cancers, and renal cell carcinoma are most commonly associated with multiple primary cancers [16]. Fourth, head and neck cancer survivors are at an increased risk of developing another cancer of the respiratory or digestive tract [17]. A “field cancerization effect” was assumed to explain this phenomenon, with carcinogens to which the organ has been exposed initiating the proliferation of numerous clones of cells [18]. Carcinogenic insults, such as tobacco and alcohol, may increase the likelihood of multiple independent malignant foci developing in the mucosa epithelium. The patient described in the present study chronically smoked tobacco and never drank alcohol. He did not receive chemotherapy or radiotherapy and did not have intense exposure to carcinogens. All of the neoplasms were not in the head and neck region, but specific cancer history was recorded in his pedigree; his father died of hepatic cancer and his mother died from lung carcinoma as well as his history of hepatitis B. We can say that the patient's stomach carcinoma presenting with a synchronous liver cancer had relation to his specific cancer history in his pedigree, whose detailed molecular mechanism remains to be elucidated. Multiplicity of primary malignancies itself does not necessarily indicate a poor prognosis, as long as adequate diagnosis and management are performed [4]. Early diagnosis may be the key risk factor affecting prognosis. At present, there was no standard treatment for multiple primary tumors. We reported the case; the patient who had stomach carcinoma presenting with a synchronous liver cancer had performed radical gastrectomy for cancer as well as liver tumor resection. Surgical excision was a useful method for multiple primary cancers; in particular, multiple primary tumor resection may be the best method.

There are some limitations in this report. First, we described only one family history investigation, which limits the ability to provide robust evidence. We should investigate more similar cases. Second, a detailed explanation for the mechanism of multiple primary tumors could not be demonstrated. We need further basic research for the detailed mechanism. Third, Follow-up time is not enough for our patient; more follow-up time is needed. Despite these limitations, we believe that the present study may improve the clinical recognition of multiple primary cancers, recognizing families at risk. Early diagnosis for additional primary malignancies should be needed for families who had specific cancer history in their pedigree.

## 4. Summary

More and more cases with multiple primary cancers have been reported in the recent literature, but mechanisms of multiple primary malignancies were unclear. There may be a great deal of risk factor. Our study suggests that family history may be a key risk factor for multiple primary tumors, which need elucidate detailed molecular mechanism. In addition, we need more family history investigation and more follow-up time in order to find more risk factors. Healthy living habits like no smoking and much more fresh vegetables and fruits on the table should be suggested. These people who have a family history of cancer also are advised to have a physical examination periodically, and those who ever had a kind of cancer, they should be followed up consanguineously. Early detection for additional primary malignancies should be needed for families who had specific cancer history in their pedigree. Early diagnosis and treatment may be the key risk factors affecting prognosis. Two or more tumor resection may be a useful method, but more follow-up time is needed.
